# Opioid Therapy in Cancer Patients and Survivors at Risk of Addiction, Misuse or Complex Dependency

**DOI:** 10.3389/fpain.2021.691720

**Published:** 2021-11-16

**Authors:** Joseph V. Pergolizzi, Peter Magnusson, Paul J. Christo, Jo Ann LeQuang, Frank Breve, Kailyn Mitchell, Giustino Varrassi

**Affiliations:** ^1^NEMA Research, Inc., Naples, FL, United States; ^2^Centre for Research & Development, Uppsala University, Uppsala, Sweden; ^3^Department of Medicine, Cardiology Research Unit, Karolinska Institutet, Stockholm, Sweden; ^4^Department of Medicine, Johns Hopkins School of Medicine, Baltimore, MD, United States; ^5^Department of Pharmacy Practice, Temple University School of Pharmacy, Philadelphia, PA, United States; ^6^Paolo Procacci Foundation, Rome, Italy

**Keywords:** cancer, opioid, opioid dependency, opioid use disorder, pain, cancer pain, opioid agonist therapy

## Abstract

A clinical conundrum can occur when a patient with active opioid use disorder (OUD) or at elevated risk for the condition presents with cancer and related painful symptoms. Despite earlier beliefs that cancer patients were relatively unaffected by opioid misuse, it appears that cancer patients have similar risks as the general population for OUD but are more likely to need and take opioids. Treating such patients requires an individualized approach, informed consent, and a shared decision-making model. Tools exist to help stratify patients for risk of OUD. While improved clinician education in pain control is needed, patients too need to be better informed about the risks and benefits of opioids. Patients may fear pain more than OUD, but opioids are not always the most effective pain reliever for a given patient and some patients do not tolerate or want to take opioids. The association of OUD with mental health disorders (dual diagnosis) can also complicate delivery of care as patients with mental health issues may be less adherent to treatment and may use opioids for “chemical coping” as much as for pain control.

## Introduction

Pain is prevalent in oncology patients. A population-based study of cancer patients using the Brief Pain Inventory (BPI) found that 55% had experienced pain in the prior week and this pain was moderate to severe (BPI ≥ 4) in 44% (1). Even in patients who received curative treatment ≥6 months earlier, moderate to severe pain was reported in 49 and 41%, respectively. This proportion increases to 75 and 70%, respectively, in cancer patients for whom further anticancer treatments are no longer feasible. Based on the Pain Management Index, pain treatment was inadequate for 42% of this population (1). The inadequate treatment of cancer pain has been further elucidated in a literature review, which reported that 43% of cancer patients had under-treated pain (2). Cancer patients may simultaneously experience both acute and chronic pain syndromes and have painful symptoms at multiple sites (3). Despite innovative new cancer treatments and advances in cancer care, pain remains widespread; since 40% of cancer patients now survive 10 or more years with “managed disease,” the need for chronic cancer pain therapy has become relevant (4). In 2019, an estimated 17 million Americans with a history of cancer were alive and this number is expected to exceed 22 million by 2030; of this number about two–thirds (67%) were diagnosed with cancer ≥5 years ago (5). Yet some of these patients are winning the battle against cancer only to face chronic pain.

Pain in cancer patients differs from pain in other groups. For one thing, cancer pain changes its characteristics, locations, and intensity frequently; postoperative pain, treatment-associated pain, and chronic forms of persistent pain. Cancer pain can worsen abruptly, may be characterized by abrupt and terrible flares, and is both psychologically as well as physically distressing (4). Worsening pain in cancer patients must be addressed promptly as it may indicate disease progression or recurrence (4). The National Comprehensive Cancer Network has issued guidelines for cancer survivors, defined as anyone who has ever had a cancer diagnosis; these guidelines advocate that survivor care should include not just screening and prevention for new or recurrent cancers but also pain management (6). Oncologists and primary care physicians may lack the expertise needed to manage chronic cancer pain, which can be complex (6). Opioid analgesia has been used to treat pain in cancer patients and has been advocated since 1986 when the World Health Organization published its three-step “pain ladder,” describing the use analgesics of increasing strength as pain intensity increased (7).

Opioids are generally effective analgesics but are associated with known and potentially treatment-limiting side effects as well as risks for opioid use disorder (OUD) (8). Pain treatment can be especially complex for patients who are actively abusing illicit drugs, who have a history of substance use disorder, or who are at elevated risk for OUD. The question about opioid analgesia (as the WHO pain ladder advocates) in patients at special risk for OUD becomes challenging, as it is not appropriate to abandon a cancer patient to pain, but it is also not prudent to expose any patient to undue risk for OUD. This has led to a decrease in opioid prescribing. According to the National Cancer Center, opioid prescribing by oncologists to cancer patients on Medicare decreased 21% from 2013 to 2017 and by 23% among other physicians, despite the fact that more cancer patients have increased life expectancy (9).

## Materials and Methods

This is a narrative review in which the authors used the PubMed, the Medline database of the National Library of Medicine, to search for literature using keywords of “cancer pain opioid risk” (658 results), “cancer pain opioid use disorder” (345 results), “cancer pain OUD” (23 results), and “cancer pain risk of addiction opioids” (105 results). The delimiter of 5 years was used since opioid prescribing has changed in the past 5 years and older articles would be less relevant to current medical understanding of opioid use and misuse. Articles not about cancer pain or opioids were excluded. The bibliographies of these articles were also used to retrieve relevant background or supplemental information.

## Results

Pain is prevalent in cancer patients, who have similar risks as the general population for OUD (10). A meta-analysis reported that pain prevalence was 39% among cancer patients who had curative treatment, 55% among those receiving anticancer treatment, and 66% of those with advanced disease, metastases, or those near end of life (11). A study from the National Veteran Health Administration (*n* = 482,688) found 6.6% of cancer patients had a comorbid diagnosis of substance use disorder and these veterans had a greater rate of medical and psychiatric disorders than those with cancer alone (12). A study of 169,162 adult cancer survivors found cancer survivors were more likely to have an opioid prescription in the past year but that opioid misuse among cancer survivors was similar to those without cancer, in other words, cancer survivors have greater likelihood of taking opioids than people without cancer but not necessarily higher rates of abuse (13). When opioid analgesics are being considered, cancer patients should be assessed for their personal risk for OUD, and the risks and benefits of opioid therapy should be discussed in a shared decision-making model. The prescribing choices for pain control must be made on an individual case-by-case basis.

### Risk Stratification for OUD

The risk factors for OUD are well-known and there are screening tools available, such as the Revised Screener and Opioid Assessment for Patients with Pain (SOAPP-R), to help stratify patients (14). Risk factors for OUD include a personal history of substance abuse (including alcoholism), a familial history of substance abuse, younger age (<65 years), male sex, certain untreated mental health conditions including depression, social or familial environments that encourage substance abuse, past incarceration, current smoking, and adverse childhood experiences (15–17). A few individual risk factors may change over time so periodic reassessment may be appropriate (18).

Assessing so-called drug-seeking aberrant behaviors in cancer patients can be challenging, because many common “red flags” have alternate explanations. Patients who actively seek opioids, particularly by product name and specific dose, may be misusing these drugs, but this action can likewise be explained by patients who live with severe pain and know exactly what works for them. While asking for higher doses of opioids to manage pain may be an aberrant drug-seeking behavior, it may also be the result of tolerance where the patient needs higher opioid doses in order to control the same amount of pain. Unusual requests, such as claiming to have lost or accidentally disposed of an opioid prescription, urgent demands for an immediate appointment, or requests for the last appointment of the day, may be signs of OUD, particularly if they occur frequently. However, an occasional request of this nature may not mean anything at all in a stressed cancer patient with a lot of cancer-related medical appointments and treatments to manage.

By the same token, these risks must be taken seriously. In a study of 1,554 cancer patients receiving opioid therapy from outpatient care at a single center from 2016 to 2018, it was reported that 19% exhibited non-medical opioid use within 8 weeks after their intake consultation. Being single or divorced, having a score on the SOAPP tool >7, experiencing greater pain severity, and taking higher doses of opioids were all risk factors for non-medical opioid use, defined as taking opioids in ways other than exactly as prescribed (19). It appears that most cancer patients taking opioids do not develop OUD, but a subset of patients is at risk.

### Patient Education, Informed Consent, Agreements

Regardless of the cancer patient's individual risk for OUD, before trialing an opioid, it is important to align the clinical objectives with the patient's expectations. For example, patients may hope to become pain free and have fully restored function and health-related quality of life, but these may not be realistic or even desirable goals. A study found that breast cancer patients were more interested in understanding their cancer pain and knowing what to expect than having that pain eliminated (20). When cancer patients cannot get the information they need, it may contribute to their illness-related distress (21). Cancer patients may be worried about how they will manage pain and may have specific analgesic outcomes in mind: restoration of function, going back to work, getting good sleep, and so on. For patients, functional goals may be more meaningful than lower pain scores. The shared decision-making model means that patient and prescriber discuss goals, weigh the risks and benefits of various pain control strategies, and then both consent to specific terms and treatments; such agreements should be put in a plain language document and periodically reviewed and, if necessary, revised (22).

While a frank discussion about the risks and benefits of opioid analgesia is appropriate for any cancer patient, it is crucial for patients at risk for OUD. Such patients should be informed about the risks of tolerance, physical dependence, opioid-associated side effects, OUD, and that opioids can be misused for “chemical coping” or recreational purposes rather than analgesic benefit (4). If the patient and clinician can build a relationship of trust, the patient may be able to discuss past substance use, current drug use, and other risk factors; however, not all patients are forthcoming in this way, particularly if they fear it may cause their cancer pain to go untreated. Guidelines for opioid therapy should be discussed with the patient and in some cases also with family and caregivers. Treatment may include periodic urine analyses, pill counts, a signed treatment agreement, instructions about how to use and store opioids, and the use of naloxone or other rescue agents (23–26).

Patients receiving long-term opioid therapy, particularly those at risk for OUD, should be under close clinical supervision. Chronic opioid use is associated with a number of adverse effects, including mental fogginess, decreased sexual drive, lower fertility, opioid-induced hyperalgesia, chronic treatment-refractory constipation, and central sleep apnea (8, 27–30). Patients should be advised about the potential for side effects, which in some cases can be managed.

In this connection, it should be noted that cancer patients face higher rates of depression, anxiety, and distress compared to the general population (31), and such conditions may exacerbate the risk for substance use disorder. In a systematic review of breast cancer studies, it appears that such psychological symptoms may be durable over many years after diagnosis (32).

### Clinician Education

Despite breakthroughs in cancer treatment, oncologists and other specialists may not be adequately trained in pain control (33) and with recent guidance from the Centers for Disease Control and Prevention (CDC) about opioid prescribing by primary care physicians (34, 35), there is increasing reticence to prescribe opioids altogether (36). Oncologists may be more focused on treating the disease than managing pain (37). A survey of oncologists (*n* = 354) found their most frequently reported barriers to adequate cancer pain management were: poor pain assessment, reluctance to prescribe opioids, and regulations surrounding opioid prescribing (38). Oncologists also reported that some patients refused opioids, even when indicated, and some hesitated to report pain, perhaps out of fear that it meant worsening prognosis (38). Only 14 and 16% of oncologists referred patients to pain specialists and palliative care specialists, respectively (38).

Prescribing opioids requires a familiarity with the various types and routes of administration of opioid analgesic products, a basic understanding of equianalgesic dosing when transitioning between opioid products, and the role of combination therapy (opioid + non-opioid) (39). Prolonged opioid exposure will result in tolerance, which requires higher doses of the opioids to maintain the same level of analgesia; tolerance is the normal and expected result of prolonged exposure to opioids (40). Tolerance may result in inadequate analgesia unless doses are increased (41). On the other hand, long-term opioid use may result in opioid-induced hyperalgesia which paradoxically lowers the pain threshold in certain patients; such patients may complain of inadequate analgesia but increasing the opioid dose worsens their pain (42). An opioid-dependent patient will develop withdrawal symptoms if the opioid is abruptly discontinued or substantially decreased; tapering programs can help a long-term opioid patient transition to opioid cessation with minimal distressing withdrawal symptoms (43).

When cancer patients present with both moderate to severe pain plus risk factors for OUD, referral to a pain specialist may be warranted. OUD is characterized by intense drug cravings, reduced understanding or appreciation of one's own behavior, dysfunctional emotional response, lack of behavioral controls, and an inability to stop taking the drugs (39). Patients who are known or strongly suspected to have active OUD are likely already taking opioids on a regular basis and are unlikely to discontinue them; some may be taking street drugs of unknown provenance and composition. Polysubstance abuse, the concomitant use of multiple drugs including street drugs, is prevalent among people with OUD (44). These opioids and possibly other drugs may or may not be adequate to control pain, but a patient with OUD might use a cancer diagnosis to obtain more or prescription opioids (45). On the other hand, an overzealous approach about restricting opioids may result in inadequate analgesia, and that, in turn, might lead to using street drugs to fight pain. People with OUD already know how to obtain opioids and can be extremely resourceful; denying them prescription opioids in no way assures they will not self-medicate. Those who have used opioids for a long time may have lower pain thresholds but higher opioid tolerance levels, meaning that an opioid-tolerant cancer patient in moderate pain may require higher doses of opioids than a similar opioid-naïve patient (46). Cancer patients who have overcome OUD or some other substance use disorder as well as the “chippers,” the slang term for occasional recreational opioid users, may be at special risk for using opioids on a more consistent basis when they are exposed to the stresses of moderate to severe pain (46). Cancer patients who are on opioid agonist therapy, using buprenorphine or methadone, are sometimes prescribed short-acting opioids to manage cancer pain in addition to their maintenance therapy, with the strategy that they will eventually be able to taper and discontinue these short-term opioids without OUD relapse (47). A case report in the literature describes a buprenorphine maintenance in a non-cancer patient who was successfully treated with tramadol 50 mg three times a day to manage orthopedic pain in addition to his buprenorphine treatment prior to surgery. He found analgesic relief with tramadol and successfully discontinued the tramadol after recovery from surgery (48). Cancer pain in patients on opioid maintenance, that is, in recovery for OUD, may be managed by adding non-opioid analgesics to synergistically supplement their maintenance opioids; increasing the maintenance opioids; or tapering off the maintenance program and taking opioid analgesics for cancer pain.

Some people who are in recovery from OUD refuse opioids, preferring to deal with pain rather than risk relapse. If non-opioid medications can control pain effectively or at least sufficiently, this may be an excellent solution. In some cases, combination therapy with a non-opioid analgesic plus adjuvants may be appropriate. Cancer pain can sometimes be managed with a procedural intervention or other technique, such as an implantable intrathecal drug pump (49, 50). Left untreated, severe or very severe cancer pain may be a risk factor for worsened outcomes, resulting in mental distress, even depression, neglected rehabilitation, social withdrawal, and diminished quality of life (46).

The population of people with OUD have a high consumption of healthcare services and disproportionately high healthcare costs (51, 52). As many as 11% of hospital patients (including but not limited to cancer patients) may have some type of active substance use disorder (53, 54). Should an individual using illicit opioids be hospitalized, opioid withdrawal symptoms can begin in 12–36 h if they have no access to opioids, although this window of time is variable as is the severity of the withdrawal symptoms (55). Thus, clinicians should be alert to withdrawal symptoms in hospitalized patients, who often do not disclose their OUD upon admission.

Clinicians may have implicit biases and suspect that a cancer patient with OUD or at risk for OUD may be manipulative and deceptive about reporting pain (56). Prescribers may fear being deceived to the point that they marginalize discussions about pain to avoid having to make judgment calls about the use of analgesics (56). By the same token, cancer patients with OUD may be fearful they will not get pain relievers at all unless they emphasize and even exaggerate their painful symptoms (57). For those reasons, a shared decision-making model should be used to discuss cancer pain, analgesic solutions, and pain management objectives (55).

It may not be automatically assumed that cancer patients with OUD desire opioid rehabilitation (56). In the setting of cancer with its potentially painful treatments and surgeries, financial stresses, and difficult prognoses and decisions, the patient with OUD may feel far too overwhelmed to embark on battling addiction at this juncture in life. While some hospitalized patients with OUD may be denied opioids and treated for withdrawal or transitioned to opioid agonist therapy, this approach vastly complicates care, particularly if the patient is facing a potentially life-threatening oncological crisis (56). For some patients with OUD, it may be helpful to prescribe opioid agonist therapy and also prescribe short-acting opioids to manage acute pain episodes (58). Short-acting opioids should not be administered in OUD patients alone, as withdrawal may occur with analgesic gaps (55). Initiating buprenorphine or methadone in a hospitalized cancer patient with OUD may facilitate patient management and prevent withdrawal symptoms, with the potential benefit that the patient may achieve recovery (55). Managing such patients out of the hospital setting is vastly different, and patients with substance use disorders have a high rate of leaving a hospital against medical advice (59, 60).

Although clinicians are increasingly confronted with cases of managing moderate to severe pain in cancer patients with OUD or at high risk for OUD, there is a paucity of literature, clinical studies, or guidance as to how to best manage treatment.

### Opioid Effectiveness

Opioids are not always effective against pain in cancer patients; indeed, some types of pain do not respond to opioids (61). Despite the fact that opioids are frequently prescribed to cancer patients, there are few high-quality randomized trials reporting on their efficacy for cancer pain management. A systematic review of the literature found fair evidence supporting the use of transdermal fentanyl for cancer patients, but poor evidence for the use of morphine, tramadol, oxycodone, methadone, and codeine (62). In a prospective, open-label, randomized controlled trial of 198 cancer patients randomized to morphine or oxycodone monotherapy, responders to the therapy were similar at 62 and 67% for morphine and oxycodone, respectively and these rates were 67 and 52% when the groups changed drugs. Thus, about 30% of cancer patients may not obtain adequate analgesia from opioid therapy; moreover, tolerability issues and side effects may further limit treatment (63). Opioid rotation from one agent to another may benefit a subset of these patients (64) in a study of 186 palliative care cancer patients, changing opioids was able to provide adequate analgesic with tolerable side effects in 96% of patients (65).

While opioids are effective analgesics, they can paradoxically cause pain to increase over time. Opioid-induced hyperalgesia is a condition in which prolonged exposure to opioids actually lowers the pain threshold and increases pain levels; pain is better controlled when opioids are discontinued (66). High doses of opioids are associated with the risk of potentially life-threatening respiratory distress (67). Opioids are not well-tolerated by all patients and while some opioid-associated side effects can be managed, others are more distressing and challenging to treat, such as opioid-induced constipation (8, 68).

### Matching Analgesic Options to the Patient

Risk stratification may be less clinically relevant in palliative care at end of life. They differ from the burgeoning group of cancer patients actively living with managed disease and concomitant pain. In general, non-pharmacological and non-opioid pain treatments should be considered as first-line therapy for most, if not all, cancer patients, regardless of the risk of OUD with the recognition that they may be ineffective for certain types of pain. For postsurgical patients and patients undergoing anticancer treatment or suffering tumor-related or other cancer pain, there may be few if any viable alternatives to opioids. Cancer patients cannot simply be relegated to deal with intolerable pain, decreased function, and a diminished quality of life, so the clinician and patient may have to determine if the benefits of opioid use outweigh the risks and how those risks can be managed. Such management strategies include close clinical monitoring, periodic patient interviews, regular urine drug monitoring, use of the prescription monitoring program, periodic pill counts, limited refills and small-quantity prescriptions. Referral to a pain specialist, an addiction medicine specialist, or psychiatric professional may be appropriate (33).

In some cases, combination drug therapy may be beneficial. There are numerous fixed-dose combination products on the market, such as acetaminophen and oxycodone or tramadol plus diclofenac, and loose-dose combinations may be selected in the form of multiple drugs (non-opioid plus small quantity of opioid) to allow for greater dosing flexibility (69, 70). In some cases, combination therapy can provide equivalent analgesia to opioid monotherapy but with substantially lower opioid doses (71). Although there are few studies that can document this finding specifically in the cancer pain population, lower opioid doses should theoretically reduce the incidence and severity of opioid-associated side effects (72).

Clinicians must be alert to the possibility for opioid diversion as, regrettably, cancer patients are often targeted by drug seekers among their friends and family, because they are potential steady sources of prescription opioids (73). Opioid prescriptions and the drugs themselves can also be readily converted to cash by those so inclined to do so.

Cancer patients should only be considered for opioid therapy after frank discussions of risks, benefits, and side effects. A plain-language patient agreement can be helpful to describe the treatment goals, the therapy, and the conditions that apply to the patient; such agreements require the prescriber to provide realistic pharmacological options to manage pain and the patient agrees not to abuse or divert these medications (74). In this context, it must be noted that pain control is only part of the issue for cancer patients with OUD. Opioid therapy for such patients may contribute to their addiction, which, in turn, can increase patient suffering, places undue stress on caregivers and family, and mask crucial symptoms of disease progression (75). In addition, cancer patients with OUD may fail to comply with medical advice and become more preoccupied with managing their pain (and sometimes mood) than fighting the disease or recovering their overall health (75).

Opioids should be administered in the lowest possible effective dose for a short time in order to trial the results. Patients should be thoroughly informed about possible side effects and therapeutic objectives should be discussed with the patient. For most patients outside palliation, an exit plan for opioid therapy rather than open-ended prescriptions is appropriate.

### Understanding Cancer Pain in Context

Pain, a biopsychosocial phenomenon, is one of the oldest and most commonly described symptom, but pain as a medical subspecialty is in its infancy. Efforts to treat pain aggressively, even calling for analgesia as a fundamental human right (76), have shifted the concept of pain as a self-reported subjective individual experience to a more objective medical category. This, in turn, led to more widespread prescribing of opioid pain relievers with concomitant increases in opioid-related morbidity and mortality as well as increases in OUD (77). Oncology patients are not the main driver of opioid misuse, diversion, and overdose, but they often urgently need opioid analgesics to manage their pain. In this context, it is important that clinicians recognize that acute and chronic cancer pain presents different patient needs and demands different prescribing choices compared to acute and chronic forms of non-cancer pain (77).

Prescribers may need to engage in frequent conversations with cancer patients to best understand their frequently changing pain characteristics and analgesic needs. Increasing pain intensity, new pain locations, or other changes in pain can signal cancer progression. Pain can also change with many aspects of the patient's living situation, familial support, financial concerns, mental health, and overall outlook, all of which may be disrupted by the cancer treatments. A clinician treating pain in a cancer patient must use a differential approach: how can the pain be effectively managed and, balanced against that, what are the risks of opioid-associated side effects, tolerance, OUD, chemical coping, hyperalgesia? (78).

### Dual Diagnosis

Substance use disorder and mental illness are strongly associated (79–81). Emotional abuse in childhood as well as a range of other adverse childhood events increases the risk for both substance use disorder and suicide (82). An individual trying to self-medicate psychic pain or mental illness using a substance may inadvertently launch a vicious cycle where the “soothing substance” produces neuroadaptive changes in the brain which form a vicious circle and drive the desire to use more substances (79). Prescribers may wish to ask patients about their own and familial histories of depression, anxiety, mental health disorders, and related diagnoses, recognizing that a cancer diagnosis can be a stressful, depressing, and anxiety-provoking event even for a person of good mental health.

Cancer is associated with mental illness as well. Major depressive disorder is up to four times more prevalent among cancer patients than in the general population (83). The psychosocial stress of cancer can increase inflammation, promote oxidative stress, depress the immune system, and initiate a dysfunction activation of the autonomic nervous system and hypothalamic-pituitary adrenal axis (83, 84). Accurate and timely diagnoses of depressive symptoms in cancer patients may allow for beneficial interventions, including such conservative first-line approaches as promoting healthy lifestyle choices, healthier diet, weight management, physical exercise, and better sleep hygiene (83, 85). Untreated depression as well as stress can contribute to cross-talk between the tumor and host cells and may potentially worsen outcomes (86).

Anxiety is likewise prevalent among cancer patients and may affect patients at any point in the course of their cancer. Benzodiazepines are sometimes prescribed for anxiety (87), but they are indicated only for short-term and carefully monitored use (88), and it is not recommended that they be taken concurrently with opioids because of the risk of potentially fatal respiratory depression (34).

A population-based retrospective cohort study from Taiwan compared cancer patients with (*n* = 1,001) and without (*n* = 4,004) schizophrenia, and determined that schizophrenic cancer patients used less opioids in the month before death (69.6 vs. 84.8%, odds ratio 0.40, 95% confidence interval, 0.34–0.48) but reasons for this remain unclear (89). Schizophrenics are often non-adherent to medical regimens, but it has been speculated that terminally ill schizophrenic cancer patients may be hypoalgesic (89). There may also be disparities in palliative care for schizophrenics.

There is a paucity of studies about mental health disorders in cancer patients and how mental health conditions might impact the cancer trajectory. People with pre-existing or new mental health conditions may be at elevated risk for OUD and may benefit from referral to psychiatric care.

### Aberrant Drug-Seeking Behaviors

Aberrant drug-seeking behaviors include asking for an opioid by name, demanding higher and higher doses, “losing” prescriptions or pills and asking for replacements, having multiple opioid prescriptions, and having multiple prescribers (23). Opioids are available as analgesics, but can be used as recreational substances or for “chemical coping” as well. In OUD patients, these objectives sometimes overlap (90, 91). Aberrant drug-seeking behaviors in cancer patients may be indicative of OUD or could reflect the patient's medical condition, where physical and emotional pain may intertwine and overwhelm the patient (92).

Pseudoaddiction is a controversial term, because not all experts differentiate it from true addiction (93). When a patient demands increased opioid analgesia, the question arises as to whether it is predicated on inadequate pain control (pseudoaddiction) or heightened craving for the opioid (true addiction), or whether such a distinction is relevant or can be drawn at all. Without a doubt, there is a blurring among the conditions formerly described as addiction, tolerance, and pseudoaddiction, and all are captured in the newer more-inclusive term OUD (41). However, it must be recognized that inadequate analgesia is possible and may be devastating for a cancer patient. Cancer patients often require opioid analgesia at relatively high doses for prolonged periods of time, which would be expected to lead to opioid dependence and opioid tolerance, in turn, necessitating higher doses of the opioid to achieve the same level of analgesia with increasing dependence on the drug, such that it cannot be discontinued without producing withdrawal symptoms (41).

At one time, nearly all cancer patients were considered appropriate candidates for opioid therapy because they likely had a terminal condition accompanied by moderate to severe—and ever-worsening—pain. Opioids may be appropriate for cancer patients, even those who survive cancer or live a reasonably functional life for many years with managed disease. Refusing to prescribe opioids to cancer patients trying to live with moderate to severe pain that is otherwise inadequately treated may represent an ethical dilemma itself. An individualized case-by-case approach is appropriate.

### The Risk of OUD in Special Populations of Cancer Pain

Younger age and male sex are both risk factors for OUD but should not preclude leaving cancer pain untreated in pediatric patients, particularly boys (94). In a study of pediatric and young adult patients with cancer administered opioids for pain control (*n* = 94), aberrant drug-seeking behaviors occurred in 12% and of those patients, and 91% had at least one risk factor for substance use disorder. This study found that in young patients, the use of multiple opioids was significantly associated with aberrant opioid-associated behaviors (*p* = 0.003) (95). The rate of OUD was not reported.

Mental illness and substance use disorder are often comorbid in a condition described earlier as dual diagnosis, but not all mentally ill patients who present with cancer will have OUD. Treating a mentally ill patient with cancer may be particularly challenging, in part because the mentally ill have a truncated life expectancy of about 10 years earlier than the general population (96). In fact, patients with severe mood disorders or schizophrenia have a life expectancy about 20 years less than the general population (97). These higher death rates are in part due to accidents and suicide but cancer and other diseases are thought to likewise contribute to this elevated mortality. Mentally ill patients may be reluctant to seek medical help, may be unable to communicate clearly about their disease, may not understand or be able to participate in their treatment, may be unable to participate in informed consent or shared decision-making, be non-adherent, and be unable to self-report pain levels (96). In addition, mentally ill patients may live in unstable or un-domiciled situations and be erratic in seeking treatments (97). Mentally ill individuals may enter the healthcare system with advanced cancer, which is associated with greater levels of pain intensity (98). The mentally ill may have poor health habits, low health literacy, and have a more difficult time accessing the healthcare system (97). In some cases, treating a mentally ill patient with cancer may require a consultation with psychiatric specialists or social workers. Many mentally ill patients are at risk with OUD or have active OUD or other substance use disorders.

Geriatric patients without a history of substance use disorder are at reduced risk for OUD, but care must be taken to avoid drug-drug interactions as many geriatric patients with cancer routinely take multiple drugs (99). Opioid dose reductions may be appropriate for older patients and care should be taken when prescribing to patients with renal dysfunction, prevalent in the elderly (100). While opioids may be appropriate for geriatric cancer patients suffering moderate to severe pain (101), cancer pain care can be challenging in patients with cognitive deficits or dementia. Mild cognitive impairment would not likely impede informed consent, shared decision-making, or accurate self-reporting of pain and symptoms, but a surrogate may be needed for more advanced forms of dementia (102). Special tools such as Pain Assessment in Advanced Dementia have been developed to assist in the care of such patients (103).

## Discussion

Opioids should not be considered, even in cancer patients, without evaluating the patient's risk for OUD, assessing their pain, and discussing with them the risks and benefits of opioid therapy. While pain control is important, even a fundamental human right (76), providing opioids to a person with OUD or at elevated risk for drug abuse may actually increase their pain (reduce the pain threshold or cause opioid-induced hyperalgesia), limit their ability to cooperate with the healthcare team, impede their efforts at recovery from OUD, reduce their quality of life, increase their global suffering, elevate their risk of potential drug-drug interactions, all while imposing undue stress on their families and caregivers (29, 49). Cancer patients should be considered for different analgesic approaches: conservative non-pharmacological therapies (if effective), complementary and alternative medicine, interventional procedures, and non-opioid pharmacological therapy, including combination regimens. Cancer patients may benefit from fixed-dose combination analgesic products, typically combining a small dose of opioid with a larger dose of a synergistically effective non-opioid, or a regimen of very low dose opioid analgesics plus adjuvant agents, such as anticonvulsants, antidepressants, muscle relaxers, and/or anti-inflammatories.

People with OUD or at high risk for OUD may develop cancer and are worthy of professional compassion and excellent care. To the greatest extent possible, prescribers and patients should have a frank discussion about pain control. Patients with OUD who present with cancer are as diverse as any other patient population: some may want to overcome their addiction and some may remain more interested in opioid use than conquering cancer. Clinicians may encounter cancer patients who, for a variety of reasons, refuse opioids, even when they are the best analgesic choice, while other cancer patients with OUD may be reluctant to even entertain any other options except opioids, even when there are other effective analgesics available.

Even when a cancer patient at elevated risk for OUD is a candidate for opioid therapy, prescribers must approach opioid analgesia with caution. On the one hand, safe and effective therapy is based on trust, so patients must have faith their clinical team will not abandon them to live with excruciating pain. On the other hand, clinicians cannot simply dispense opioids to certain patients without exposing them to risks that may be worse than pain. For instance, cancer patients with active OUD who are prescribed opioids may neglect their health, be non-adherent with cancer treatments, engage in unhealthy behaviors, and have overall worse quality of life. People with active OUD have often made opioids the overwhelming focus of their life, and this may distract them from their fight against cancer.

Cancer patients with a history of OUD on current opioid agonist therapy, typically with buprenorphine or methadone, are complex cases. An initial consultation with the other prescriber is appropriate and possibly an addiction medicine specialist should be added to the clinical team. In some situations, the dose of the agonist (opioid) can be temporarily increased; in other cases, non-opioid analgesics should be considered first to synergistically supplement the opioid for analgesic benefit. Some patients may be able to maintain the opioid reocvery program and take short-acting oral opioids to manage acute pain. It is difficult to set forth a one-size-fits-all strategy in that much depends on the patient's personality and mental health, age, the type and extent of cancer, the cancer treatments, the prognosis, and the patient's family and social situation.

## Conclusion

As more and more cancer patients survive the disease or live well for many years with managed disease, the issue of long-term analgesia becomes important. Cancer patients may be at risk for OUD; indeed, some people with cancer present with active use of illicit opioids. Managing these patients requires an individualized approach. There are tools to help stratify patients at risk for OUD. At that point, the type of cancer, prognosis, pain treatment options, and the patient's individual characteristics must be incorporated in the evaluation. It is crucial that cancer patients, even with active OUD, not be abandoned to suffer excruciating pain or be denied good medical care, but opioid prescribing is not appropriate in all patients. For some cancer patients, opioids may actually increase pain and suffering, lower quality of life, and cause more suffering. On the other hand, there are cancer patients with OUD who may need opioids to manage their cancer-related pain. Clinicians should establish good rapport with patients, frankly discuss the risks and benefits of opioid therapy, re-evaluate these patients regularly, and individualize analgesic strategies on a case-by-case basis.

## Author Contributions

JP was invited to prepare this article and provided the concepts, the structure, and clinical recommendations, reviewed the article, and provided bibliographic support. PM reviewed the manuscript and provided critical analysis of the articles and how the content was organized. JL is a medical writer who did the literature searches, prepared the bibliography, and edited the manuscript for publication. FB reviewed the articles, selected particularly relevant articles for inclusion, and reviewed the final manuscript. PC and KM reviewed the manuscript and offered critical comments on how to organize the text. GV worked on the paper concepts and bibliographic search strategies, reviewed the literature, helped interpret the body of work, and reviewed the final manuscript in detail. All authors contributed to the article and approved the submitted version.

## Conflict of Interest

JP and KM were employed by company NEMA Research, Inc. The remaining authors declare that the research was conducted in the absence of any commercial or financial relationships that could be construed as a potential conflict of interest.

## Publisher's Note

All claims expressed in this article are solely those of the authors and do not necessarily represent those of their affiliated organizations, or those of the publisher, the editors and the reviewers. Any product that may be evaluated in this article, or claim that may be made by its manufacturer, is not guaranteed or endorsed by the publisher.
